# 0730. Plasma endocan levels are associated with endothelial dysfunction during experimental human endotoxemia

**DOI:** 10.1186/2197-425X-2-S1-P52

**Published:** 2014-09-26

**Authors:** LT van Eijk, LAE Cox, BPC Ramakers, MJ Dorresteijn, J Gerretsen, M Kox, P Pickkers

**Affiliations:** Intensive Care Medicine, Radboud University Medical Center, Nijmegen, Netherlands; Radboud Institute for Infectious Diseases, Nijmegen, Netherlands; Anesthesiology, Pain and Palliative Medicine, Radboud University Medical Center, Nijmegen, Netherlands

## Introduction

The endothelium plays a central role in pathophysiology of sepsis. Systemic inflammation results in endothelial dysfunction, contributing to the development of shock and multiple organ dysfunction. However, an easy to obtain, early, and clinically applicable marker of endothelial dysfunction is currently not available. Such a marker could guide early and targeted therapy such as selective iNOS inhibition. Endocan is a soluble proteoglycan secreted by endothelial cells in response to pro-inflammatory cytokines, bacterial endotoxins, and angiogenic factors, and enhances microvascular permeability. Plasma endocan levels are increased in septic patients, and have been shown to correlate with sepsis severity and mortality. However, the direct relationship between endocan and endothelial function has not yet been investigated in humans *in vivo*.

## Objectives

To investigate the kinetics of plasma endocan levels during human endotoxemia and to examine their relation with inflammation-induced endothelial dysfunction.

## Methods

Seventeen healthy male volunteers were subjected to experimental endotoxemia (infusion of 2 ng/kg *E.Coli* lipopolysaccharide [LPS]). Plasma levels of inflammatory cytokines (TNF-α, IL-6, IL-10, and IL-1RA), endocan, ICAM, and VCAM were measured at T=0, 0.5, 1, 1.5, 2, 4, 6 and 8 hrs after LPS infusion. Both before and 4 hrs after LPS administration, endothelial function was assessed by determination of the vasodilatory response of forearm blood vessels to the incremental intra-arterial infusion of endothelium-dependent (acetylcholine) or endothelium-independent (nitroglycerine/nitroprusside) vasodilators using venous occlusion plethysmography. Furthermore, correlations between the increases in plasma endocan, ICAM, and VCAM levels, and the endotoxemia-induced changes in vasodilatory responses were explored.

## Results

**Figure 1:**
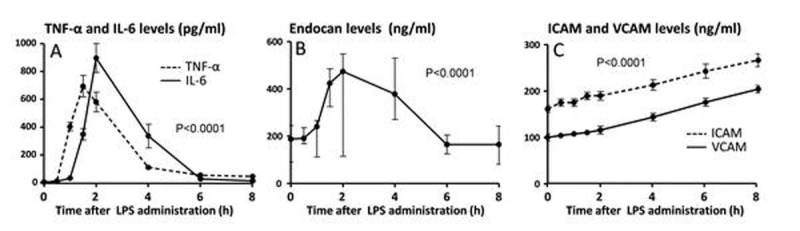
**Plasma concentrations of cytokines TNF-α and IL-6 (A), endocan (B), and ICAM and VCAM (C) during endotoxemia.** Data in panels A and C are expressed as mean with SEM, and analyzed using repeated measures one-way ANOVA (P-values apply to all indicated parameters). The non-depicted anti-inflammatory cytokines IL-10 and IL-1RA peaked at T=3 hrs (702 ± 183 pg/ml and 7332 ± 759 pg/ml, respectively, both P<0.0001). Data in panel B are expressed as median with interquartile range, and analzed using Friedman test.

**Figure 2:**
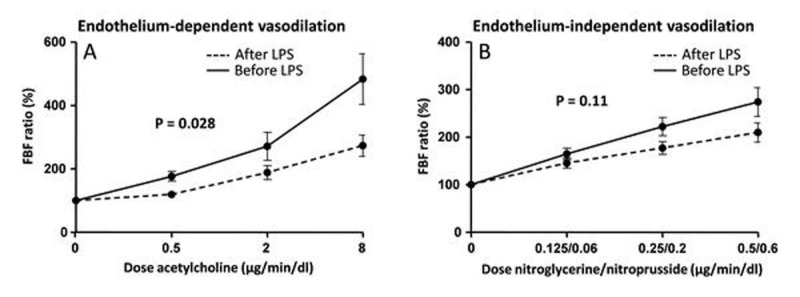
**Forearm blood flow (FBF) ratios in response to the acetylcholine (A) and nitroglyverine/nitroprusside (B) before and during endotoxemia.** FBF ratios were calculated by dividing the flow in the experimental arm by the concurrent flow in the non-infused (control) arm, to adjust for systemic changes in flow. At baseline this ratio was set at 100%. Data are expressed as mean with SEM. p-values indicate difference between FBF ratios before and after LPS administration calculated using repeated measures two-way ANOVA (interaction term).

Plasma levels of all measured cytokines, endocan, ICAM, and VCAM concentrations significantly increased after LPS administration (Figure 1). LPS administration resulted in a significantly blunted response of forearm vasculature to acetylcholine (P = 0.028, Figure 2A), whereas the response to nitroglycerine/nitroprusside was not significantly affected (P = 0.11, Figure 2B). Furthermore, there was a significant correlation between the increase in plasma endocan levels and the attenuation of vasodilatory responses to acetylcholine (Pearson's r = -0.49, P = 0.049). No correlation existed between plasma levels of ICAM or VCAM and the attenuation of the acetylcholine-induced vasodilatory response.

## Conclusions

Endocan levels are related to endothelial dysfunction during systemic inflammation in humans *in vivo*. Therefore, it may prove to be a suitable marker for the early identification of endothelial dysfunction and could guide targeted therapy.

